# Using High-Power UV-LED to Accelerate a Decatungstate-Anion-Catalyzed Reaction: A Model Study for the Quick Oxidation of Benzyl Alcohol to Benzoic Acid Using Molecular Oxygen

**DOI:** 10.3390/mi12111307

**Published:** 2021-10-25

**Authors:** Mamoru Hyodo, Hitomi Iwano, Takayoshi Kasakado, Takahide Fukuyama, Ilhyong Ryu

**Affiliations:** 1 Organization for Research Promotion, Osaka Prefecture University, Sakai, Osaka 599-8531, Japan; iwano@c.s.osakafu-u.ac.jp (H.I.); t_kasakado@c.s.osakafu-u.ac.jp (T.K.); 2 Department of Chemistry, Osaka Prefecture University, Sakai, Osaka 599-8531, Japan; fukuyama@c.s.osakafu-u.ac.jp; 3 Department of Applied Chemistry, National Yang Ming Chiao Tung University (NYCU), Hsinchu 30010, Taiwan

**Keywords:** molecular oxygen, oxidation, high power UV-LED, benzyl alcohol, benzoic acid

## Abstract

High-power UV-LED irradiation (365 nm) effectively accelerated the decatungstate-anion-catalyzed oxidation of benzyl alcohol **1** to benzoic acid **3** via benzaldehyde **2**. As the power of the UV-LED light increased, both the selectivity and yield of benzoic acid also increased. The reaction was finished within 1 h to give **3** in a 93% yield using 2 mol% of decatungstate anion catalyst. The combination of a flow photoreactor and high-power irradiation accelerated the oxidation reaction to an interval of only a few minutes.

## 1. Introduction

**Figure 1:**
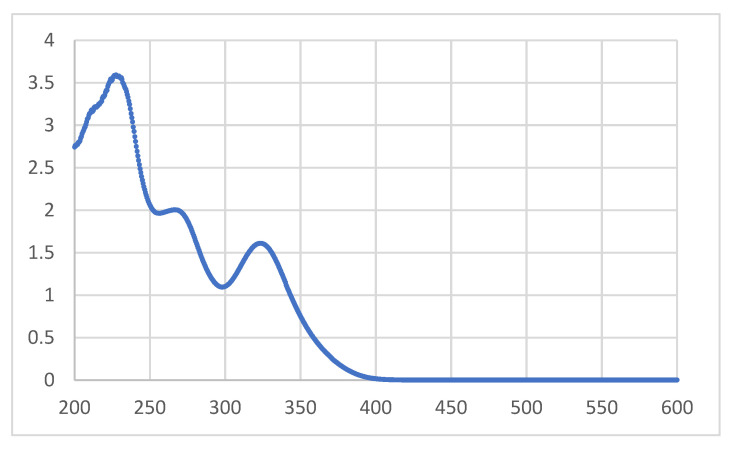
UV-VIS Spectrum of decatungstate anion in acetonitrile (1.1 × 10^−4^ M, TBADT (Bu_4_N)_4_W_10_O_32_).

Decatungstate anion (W_10_O_32_^4−^), when photo-excited, catalyzes C(sp^3^)-H functionalization via hydrogen atom transfer (HAT) to create alkyl radicals [1,2,3,4,5]. When molecular oxygen or oxidizing reagents are present, decatungstate catalyzes the oxidation of C(sp^3^)-H bonds [6,7,8,9,10,11]. The UV-VIS spectrum of decatungstate anions [12] is featured in Figure 1. To accomplish photo-irradiation, a xenon lamp is frequently used. Recent work has shown, however, that a low-power blacklight (15 W, 352 nm) some other light sources around 390 nm can also affect C-H alkylation [13]. In the pursuit of improved photo-efficiency, decatungstate-catalyzed reactions were conducted in flow using thin microchannels, which contributed to shortening of the irradiation time [14,15,16,17].

**Scheme 1:**
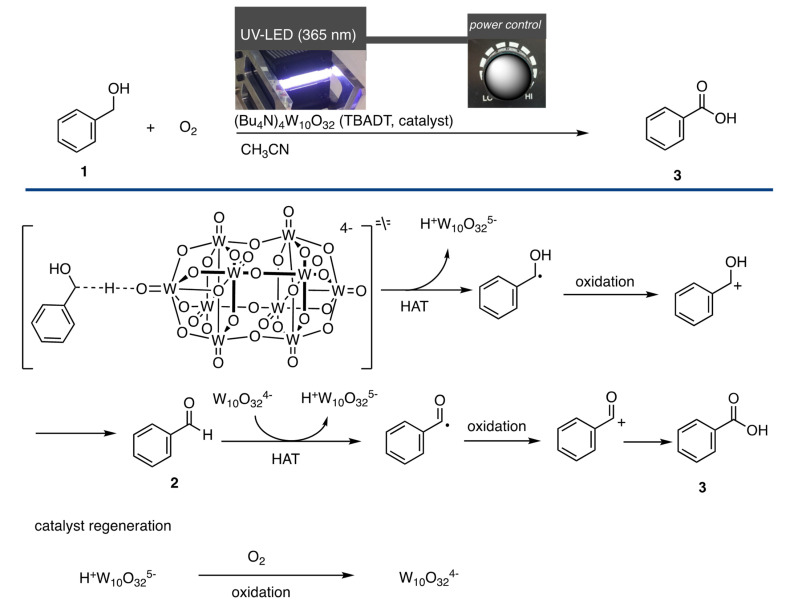
Improving the catalytic efficiency of decatungstate-anion-catalyzed oxidation using molecular oxygen via high-power UV-LED.

Despite much work dealing with photocatalytic oxidation using decatungstate anion as the catalyst, only a few studies have focused on accelerating the reaction via intense irradiation from a light source, ever since the early efforts by Hill [18,19,20]. That situation motivated us to investigate how the decatungstate-anion-catalyzed oxidation would be affected when powered by photo-irradiation equipment. We focused on catalytic oxidation using a decatungstate anion and molecular oxygen [21,22,23,24,25] under varied photo-irradiation conditions. For this study, we used a Photo System U-1, which is composed of 365 nm UV-LED light (Kyocera G-5A), a controller, and a power supply, which provides irradiation power that is adjustable in a range from 60 to 480 W. Scheme 1 illustrates the model oxidation reaction of **1**, which leads to benzoic acid **3** via benzaldehyde **2**, as well as the proposed mechanism. In this reaction, hydrogen atom transfer (HAT) by photo-excited decatungstate ion triggers the oxidation. Benzyl alcohol has only one type of C(sp^3^)-H bond available for the HAT process and is frequently used for oxidation as a model compound [26,27,28]. Accumulated H^+^W_10_O_32_^5−^ is oxidized by molecular oxygen to recover W_10_O_32_^4−^. Pleasingly, we were able to find that the reaction was complete within 1 h when high-power irradiation by 480 W-irradiation was employed, giving benzoic acid **3** in both excellent selectivity and yield.

## 2. Experimental

### 2.1. General Information

^1^H NMR spectra were recorded using Spinsolve Ultra 60 (60 MHz) spectrometers in CD_3_CN, which were referenced at 0.00 ppm for tetramethylsilane. Chemical shifts are reported in parts per million (δ). Splitting patterns are indicated as follows: br, broad; s, singlet; d, doublet; t, triplet;, m, multiplet. GC analysis was performed on a Shimadzu GC-2014 instrument equipped with an FID detector using a J&W Scientific DB-1 column under the following conditions: initial oven temperature was held at 50 °C for 5 min; the first ramp was 10 °C/min to 250 °C, which was held for 5 min. UV-visible absorption spectra were measured by V-630 Spectrometer (JASCO). The detector used for the measurement of optical density was a UV power meter C9563_H9958-01 purchased from Hamamatsu Photonics. Benzyl alcohol (**1**), sodium tungstate, and tetrabutylammonium bromide were purchased from Nacalai Tesque. TBADT was prepared according to the reported procedure [3]. The blacklight was purchased from Toshiba (Tokyo, Japan). The photo system UV-LED (MiChS UV-LED-S equipped with Kyocera G5A (365 nm, 60–480 W)), the MiChS L-1 flow system, and T-shape mixer MiChS α400 were purchased from MiChS Inc (Osaka, Japan): http://www.michs.jp (accessed on 24 October 2021).

### 2.2. Typical Procedure for the Oxidation of Benzyl Alcohol ***1***

Benzyl alcohol **1** (0.25 mmol, 25 mg) and TBADT (0.005 mmol, 15 mg) were added to a 15 mL glass tube along with a solvent (CH_3_CN, 0.6 mL) and equipped with an O_2_ balloon. The mixture was stirred at room temperature and irradiated either by a blacklight or by a UV-LED. After the reaction, Et_2_O was added to the reaction mixture and filtered to remove the precipitated TBADT. An aliquot of the solution then was applied to GC analysis.

### 2.3. The Procedure for the Isolation of Benzoic Acid ***3***

Benzyl alcohol **1** (1.0 mmol, 108 mg) and TBADT (0.02 mmol, 60 mg) were added to a 50 mL glass tube along with a solvent (CH_3_CN, 2.4 mL) and equipped with an O_2_ balloon. The mixture was stirred at room temperature for 30 min and irradiated by the UV-LED (365 nm, 480 W). After the reaction, Et_2_O was added to the reaction mixture and filtered to remove the precipitated TBADT and concentrated to dryness. The crude product (122 mg) was recrystallized from H_2_O to give a white crystalline of benzoic acid **3** (100 mg, 81% yield).

### 2.4. The Procedure for the Flow Oxidation of Benzyl Alcohol ***1***

The flow oxidation reaction was carried out using a MiChS UV-LED-S photo-system and MiChS L-1 photo-microreactor, which had a single-lane channel (2 mm in width, 1 mm in depth, 3 m in length, total volume 6 mL) covered with quartz. This photoreactor was irradiated by UV-LED (365 nm, 480 W). An acetonitrile solution containing benzyl alcohol (55 mM) and TBADT (1.1 mM) was prepared and placed in a syringe (SGE syringe, Trajan Scientific). Oxygen gas was also taken in a syringe. These solutions were pumped into a MiChS L-1 photo-microreactor through a MiChS α400 micromixer using a syringe pump at rates of 0.1 mL/min (reaction solution) and 1.1 mL/min (oxygen), respectively (residence time: 5 min). The reaction mixture eluted from the outlet was discarded for the first 15 min and the subsequent portion was collected for 15 min. The collected reaction mixture was treated with Et_2_O, filtered via a celite pad, and subjected to GC analysis to determine the yield.

## 3. Results and Discussion

**Figure 2:**
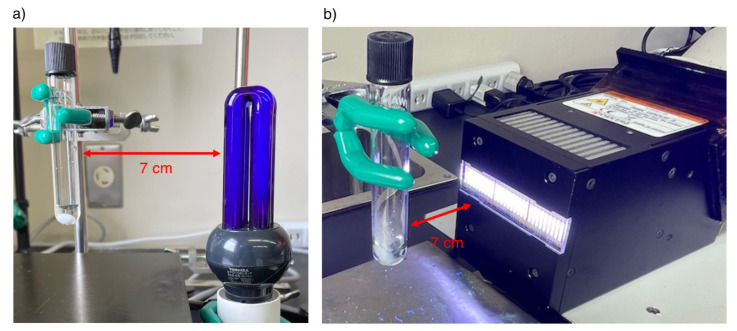
Photo-irradiation setup: (**a**) test tube reactor irradiated by a low-power blacklight (352 nm); (**b**) test tube reactor irradiated by a high-power UV-LED-S (365 nm).

The photo-reaction setup is featured in Figure 2. A test tube reactor with a screw cap (diameter 15 mm, length 10 cm) was used for the photo reaction. A low-power blacklight (352 nm, 15 W) was acquired from Toshiba (Figure 2a). A high-power UV-LED system (365 nm, (Figure 2b) was acquired from MiChS.

The oxidation of benzyl alcohol 1 (0.25 mmol) was carried out in the presence of a catalytic amount of tetrabutylammonium decatungstate (TBADT, 15 mg, 2 mol%) in acetonitrile (0.6 mL) under atmospheric pressure of O_2_. The reaction products were analyzed by GC and the results are summarized in Table 1. We started with an oxidation reaction of 1 using a test tube (Pyrex, diameter size: 15 mm) and a blacklight. The reaction using a blacklight was sluggish, and after 1 h, it gave only a 21% yield of benzaldehyde 2 and a trace amount of benzoic acid 3 (Table 1, entry 1). A similar reaction was carried out for 6 h, which resulted in 49% yield of benzaldehyde and 22% yield of benzoic acid (Table 1, entry 2). The overnight reaction (20 h) gave benzoic acid 3 as a principal product (Table 1, entry 3). Then, we switched to UV-LED, for which we applied 120, 300, and 480 W irradiation for the model reaction. After 1 h of UV-LED irradiation at 120 W, the reaction proceeded much more efficiently than the blacklight irradiation had and yielded 38% of benzaldehyde and 54% of benzoic acid (Table 1, entry 4). When we employed irradiation at 300 W, benzyl alcohol **1** was completely consumed after 1 h and a 31/69 mixture of benzaldehyde **2** and benzoic acid **3** was formed (Table 1, entry 5). Gratifyingly, 480 W irradiation provided 93% of benzoic acid **3** (Table 1, entry 6). In a separate experiment, we measured the optical intensity of the light source. Although the short distance of 7 cm was too close to measure the optical densities of UV-LED when the space was 18 cm, we could count them to be 51.6 (120), 80.8 (300), and 93.7 (480) mW/cm^2^ (V), respectively. Using these data, we estimated the optical intensities for 7 cm distance to be 350 (120), 535 (300), and 638 (480) mW/cm^2^ (V), respectively, which were two orders of magnitude larger than 1.8 mW/cm^2^ of 15 W blacklight, as measured with an aluminum foil wrapping. The temperatures of the reaction mixtures were 38 °C (blacklight) and 60 °C (UV-LED), which may have affected the solubility of oxygen in each case.

**Figure 3:**
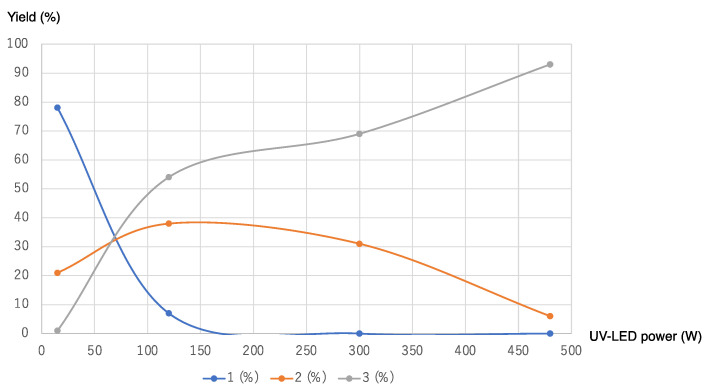
Effect of irradiation power for a 1 h reaction of **1** with O_2_.

The relationship between irradiation power and reaction progress is summarized in Figure 3. Since recent work used a light wavelength of ca. 390 nm for decatungstate-anion-catalyzed reactions [29,30,31,32]. We also tested other wavelengths of 385 and 395 nm for the irradiation of decatungstate catalyst and the reactions also proceeded well to give almost the same results compared as for irradiation at 365 nm (Table 1, entry 7 and 8). After 30 min of irradiation, benzaldehyde **2** was nearly consumed to give 92% of benzoic acid together with a small amount of benzaldehyde **2** (Table 1, entry 9). Compared with blacklight irradiation, the reaction period was shortened at least 20-fold via the use of the UV-LED system. When irradiation was stopped after 10 min, benzaldehyde 2 was formed in 44% as the sole oxidation product with 54% of benzyl alcohol 1 remaining (Table 1, entry 10), which suggested that the oxidation leading to 3 proceeds stepwise via the initial formation of **2** [33]. In the open-air reaction or in the absence of a TBADT catalyst and under 480 W of irradiation for 1 h, the reaction also proceeded and resulted in the formation of a mixture of benzaldehyde **2** (77% and 36%) and benzoic acid **3** (11% and 25%) (Table 1, entry 11 and 12). These results suggest that air also acts as an oxidant and a parallel non-catalytic mechanism exists to push the photo-oxidation of 1 [34,35,36]. We also examined the scalable photo-oxidation and 100 mg (0.81 mmol, 81%) of benzoic acid **3** was isolated after recrystallization (see Experimental 2.3).

**Figure 4:**
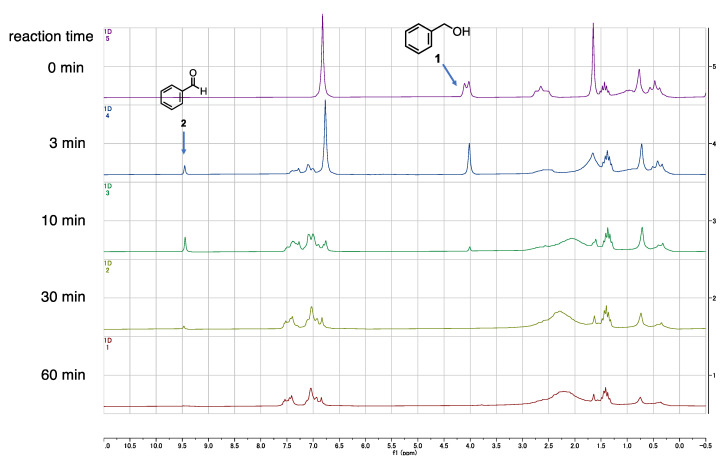
Stacked ^1^H NMR spectra (60 MHz) by time function.

**Figure 5:**
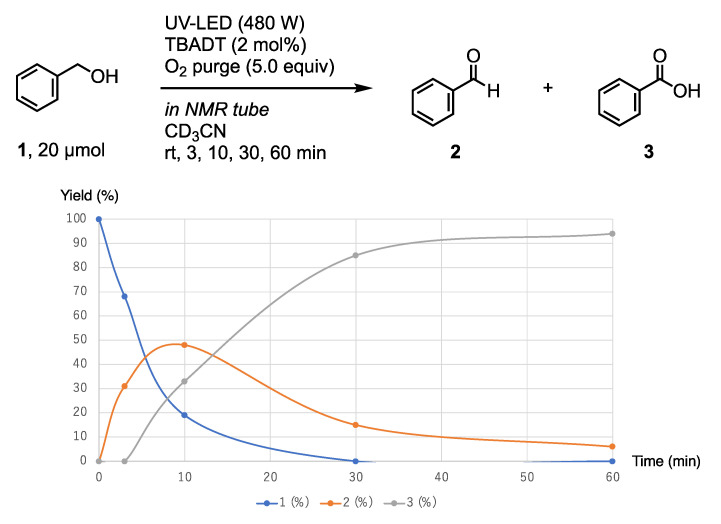
Time course for the oxidation of **1** to **2** and **3** via experiments using an NMR tube and irradiation by UV-LED at 480 W.

To monitor the reaction quickly by NMR (60 MHz, Spinsolve, Magritek), we then examined this photoreaction using an NMR tube (Pyrex, diameter size: 5 mm) as the reaction vessel. TBADT was added (1.5 mg, 2 mol%) to a solution of acetonitrile-d_3_ (0.6 mL) containing benzyl alcohol **1** (0.02 mmol). Under an atmosphere of O_2_ gas, the NMR tube was irradiated with UV-LED light (480 W) at room temperature. With irradiation, the color of the solution immediately turned blue. We used ^1^H NMR measurements at 3, 10, 30, and 60 min to monitor the reaction course (Figure 4) and the yields are plotted as a function of time in Figure 5.

**Scheme 2:**
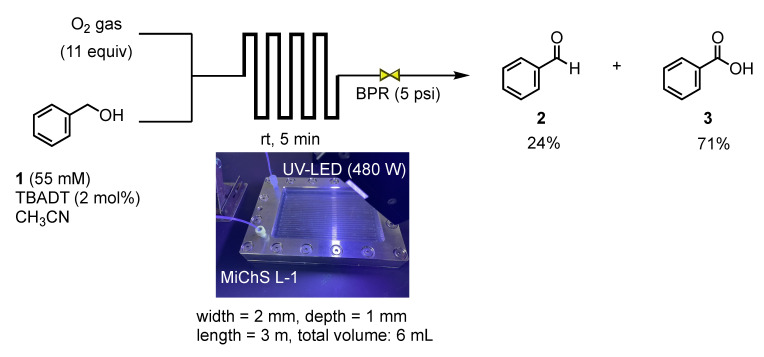
Decatungstate-anion-catalyzed microflow oxidation of benzyl alcohol **1** using O_2_ and irradiation with UV-LED (480 W).

With the efficient conditions using high-power UV-LED irradiation established, finally we set out to examine a flow oxidation reaction [37]. Using a photo flow reactor (MiChS L-1 (channel sizes: width = 2 mm, depth = 1 mm, length = 3 m, total volume 6 mL), an acetonitrile solution of benzyl alcohol (1, 55 mM) and TBADT (2 mol%) was mixed with molecular oxygen (11 equiv) using a MiChS α400 mixer (T-shape mixer with 400 µm inner diameter, supplied from MiChS, Inc.) and introduced into a microchannel of the photo flow reactor equipped with a back pressure regulator (5 psi). When irradiation using UV-LED (480 W) was carried out with a residence time of 5 min, all benzyl alcohol was consumed and a mixture of 24% benzaldehyde and 71% benzoic acid was obtained (Scheme 2).

This study was focused on the acceleration of decatungstate-anion-catalyzed C-H functionalization via the oxidation of benzyl alcohol **1** with molecular oxygen as a model. Ultimately, the reaction was dramatically improved by the powerful irradiation of the light source [38]. On the contrary, the oxidation reaction of benzyl alcohol **1** using a 15 W blacklight irradiation (352 nm) was very sluggish and gave a low yield of benzaldehyde **2**, despite the small reaction scale (0.25 mmol). After 30 min of UV-LED (365 nm) irradiation at the maximum power of 480 W, the reaction produced the desired benzoic acid **3** in high yield. These batch experiments were useful in the design of a continuous flow reaction system for these decatungstate-anion-catalyzed oxidation reactions. Indeed, by using a flow setup with a MiChS L-1 photo-flow reactor and the same exposure to a MiChS UV-LED-S photo-irradiation system, the oxidation reaction of benzyl alcohol **1** proceeded in 5 min of residence time to give benzoic acid **3** in a 71% yield. We believe that the combination of high-power irradiation and a thin flow reactor would be highly useful for the acceleration of decataungstate anion catalysis not only oxidation but also other reactions such as C–H functionalization, and research along this line is now being actively pursued in our laboratory.

## Figures and Tables

**Table 1:** Oxidation of benzyl alcohol **1** by molecular oxygen with a catalytic amount of TBADT under photo-irradiation.

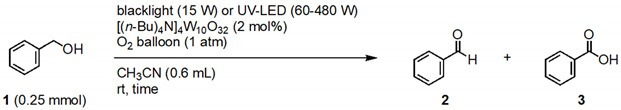						
Entry	Wavelength (nm)	Irradiation Power (W)	Time (min)	1 (%)	2 (%)	3 (%)
1 ^a^	352	15 (blacklight)	60	78	21	1
2 ^a^	352	15 (blacklight)	360	28	49	22
3 ^a^	352	15 (blacklight)	1200	0	3	97
4	365	120 (UV-LED)	60	7	38	54
5	365	300 (UV-LED)	60	0	31	69
6	365	480 (UV-LED)	10	15	18	67
7	385	480 (UV-LED)	10	13	18	69
8	395	480 (UV-LED)	10	20	24	56
9	365	480 (UV-LED)	30	0	7	92
10	365	480 (UV-LED)	60	0	6	93
11 ^b^	365	480 (UV-LED)	60	11	77	11
12 ^c^	365	480 (UV-LED)	60	39	36	25

^a^ Tube reactor and blacklight were wrapped with aluminum foil; ^b^ Air was used; ^c^ Without decatungstage catalyst.
